# Impacts of Delivery Mode and Maternal Factors on Neonatal Oral Microbiota

**DOI:** 10.3389/fmicb.2022.915423

**Published:** 2022-06-27

**Authors:** Tiansong Xu, Lihuang Yan, Bohui Sun, Qi Xu, Jieni Zhang, Wenhui Zhu, Qian Zhang, Ning Chen, Guoli Liu, Feng Chen

**Affiliations:** ^1^Central Laboratory, Peking University School of Stomatology, Beijing, China; ^2^Department of Obstetrics, Peking University People’s Hospital, Beijing, China; ^3^Department of Gastroenterology, Peking University People’s Hospital, Beijing, China

**Keywords:** neonatal oral microbiota, development phenotype, maternal factors, mode of delivery, 16S rRNA gene sequencing

## Abstract

**Objectives:**

Initial oral microbial colonization has complicatedly interacted with growth and development. The aim of our study was to discover links between oral microbiota community structure and mode of delivery, maternal factors, such as systemic diseases, abortion history, and pregnancy complications.

**Methods:**

A total of 177 pregnant women and their neonates were enrolled at Peking university people’s hospital. We collected oral samples, medical history, and development phenotype and used a 16S rRNA gene sequence to analyze microbial diversity at all taxonomic levels, network structure, and metabolic characteristics.

**Results:**

*Firmicutes*, *Proteobacteria*, and *Actinobacteriota* were the most predominant bacteria of neonatal oral samples among these phyla. Alpha-diversity of pregnant women with gestational diabetes mellitus (GDM), abortion history, and without immune diseases was higher than in control groups, and no significant dissimilarity in beta-diversity was observed between different maternal factors. Obvious separation or trend failed to be seen in different development phenotype groups. Besides, *Oscillospirales* were significantly more abundant in a natural delivery group than in the cesarean section group.

**Conclusion:**

Our study indicated that maternal factors and mode of delivery influenced the oral microbial structure, but longitudinal studies were indispensable for capturing the long-term effects on neonatal development phenotype and oral microbiota.

## Introduction

About 100 trillion microbial cells inhabited the human body, forming a network of complex ecosystems. They were critical to the host’s health, and the imbalance of microbiota was relevant to many human diseases (White et al., 2020). However, the properties of the microbiota of humans were largely a black box (Cho and Blaser, 2012). And human symbiotic microbiota had a vital effect on the early and long-term neonatal physiological consequences (Al-Shehri et al., 2016; Fujimura et al., 2016; Yang et al., 2022). For instance, the dysbiosis of the neonatal gut microbiome encouraged immune cell dysfunction related to childhood atopy (Fujimura et al., 2016). Microbial community stability might potentially act as biomarker for infant health (Al-Shehri et al., 2016).

Accordingly, increasing awareness of human microbes would be instrumental in childhood development and immunity basically. Moreover, researchers were paying more attention to the relationship between human macro and micro fields, especially human development phenotypes. Recently, Li et al. (2022) maintained that fingerprint patterns were genetically related to hand and finger proportions. Nevertheless, studies on human microbiota and developmental phenotypes were rare.

The initial microbiota of newborns has changed dynamically in the gut during pregnancy and childhood development by complex factors (Younge et al., 2019). Many previous studies focused on the effect of mode of birth and infant nutrition on neonatal microbiota (Ruiz et al., 2019; Kageyama et al., 2022). A system review confirmed that maternal breastfeeding did not appear to affect oral mycology, but vaginal delivery seemed to facilitate oral yeast colonization in early life (Azevedo et al., 2020).

In addition, the maternal state also influenced the colonization of neonatal microbiota (Tuominen et al., 2019; Ishimwe, 2021). Aerobic vaginitis and abnormal vaginal microbiota were significantly associated with a higher incidence of premature rupture of membranes (PROM) (Leitich et al., 2003; Han et al., 2018), as endogenous bacteria and lower genital tract infections could ascend into the upper genital tract and uterus through the vagina and cervical canal (Hansen et al., 2014). PROM was associated with instability of vaginal bacterial community structure during pregnancy, reduced *Lactobacillus* spp. abundance and increased relative abundance of potentially pathogenic species, namely, *Prevotella*, *Peptoniphilus*, *Streptococcus*, and *Dialister* (Paramel Jayaprakash et al., 2016; Brown et al., 2019). Gestational diabetes mellitus (GDM), one of the most common pregnancy complications, was a disease of abnormal glucose tolerance, leading to a series of short- and long-term complications (Olabi and Bhopal, 2009; Khambule and George, 2019; Wu et al., 2021). Moreover, maternal diabetes status might cause microbiota dysbiosis in the meconium of newborns (Hu et al., 2013). Dysbiosis in pregnancy was bound up with increased pathogenic microbes and metabolites that caused hypertension and adverse pregnancy outcomes (Ishimwe, 2021). Moreover, the presence of bacteria in the placental tissues of a subset of women with pre-eclampsia supported the role of bacteria in the multifactorial cause of pre-eclampsia (Amarasekara et al., 2015). However, the complicated interaction between maternal factors and neonatal microbiota, the development phenotype remained enigmatic. Also, periodontitis was demonstrated to be linked to the risk of adverse pregnancy outcomes, since periodontal pathogens and inflammatory molecules could diffuse through the bloodstream leading to the inflammation of the placenta (Gare et al., 2021).

In the study, we collected and analyzed oral microbiome samples and development phenotype from 177 newborns, and maternal factors and mode of delivery from their mother. We performed 16S rRNA gene sequencing to analyze microbial diversity at all taxonomic levels, network structure, and metabolic characteristics, to illustrate the influence of maternal factors and delivery mode on neonatal oral microbiota and phenotype.

## Materials and Methods

### Study Participants

One hundred and seventy-seven pregnant women and neonates were enrolled at Peking University People’s Hospital from August to November 2019. The medical history of pregnant women was diagnosed by specialized doctors. Informed consent was signed by parents or guardians for all participants. The procedures and experiments in this study were approved by the medical ethics committee of the study hospital (2019PHB140-01).

### Sample Collection

The medical researcher collected oral samples cautiously by swabbing neonatal oral buccal mucosa with sterile swabs when neonates were delivered within 3 days. Then the sterile swabs containing the samples were sealed in PBS buffer and stored in an −80^°^C freezer until further processing.

### 16S rRNA Amplicon Sequencing and Pre-processing

The microbiota composition of the oral samples was analyzed using 16S rRNA amplicon sequencing. DNA was extracted from samples with the use of the TIANamp Swab DNA Kit (Tiangen Biotech, Beijing, China) according to the manufacturer’s instructions. The 16S rRNA V3–V4 variable region was amplified using forward primer 338F (5′-ACTCCTACGGGAGGCAGCAG-3′) and reverse primer 806R (5′-GGACTACHVGGGTWTCTAAT-3′). Purified amplicons were pooled in equimolar and paired-end sequenced on an Illumina MiSeq PE300 platform (Illumina, San Diego, CA, United States) according to the standard protocols by Majorbio Bio-Pharm Technology Co., Ltd. (Shanghai, China).

Raw sequencing reads of the 16S rRNA gene sequences were quality filtered and analyzed using QIIME2 (version 2021.4)^1^ (Bolyen et al., 2019). We removed barcode and primer, controlled quality, corrected amplicon sequence data, and dereplicated with DADA2. The sequence data were trimmed at 20–275 bp to obtain high-quality sequences. Taxonomic identification of bacteria was determined on the Silva reference databases (Quast et al., 2013).

Then, we derived a phylogenetic tree and calculated alpha-diversity and beta-diversity through QIIME 2. Alpha-diversity was included Observed_features, Shannon_entropy, pielou_evenness, and Faith’s Phylogenetic Diversity (Faith_PD). The Bray–Curtis, Jaccard, unweighted and weighted UniFrac distance metrics, and principal component analysis (PCA) were used to describe and visualize beta-diversity.

Functional predictions of 16S rRNA data were inferred using the R package Tax4Fun (Aßhauer et al., 2015). A closed reference feature table from the QIIME2 with a SILVA database release version 132 was applied to the analysis. Tax4Fun could predict and survey the functional capabilities of microbial communities based on 16S rRNA sequencing data, and provide a good approximation to functional profiles gained from metagenomic shotgun sequencing means.

### Statistics and Analysis

Continuous numerical variables were presented as means ± SDs if the data were in accord with normal distribution, median (upper quartile and lower quartile) if not, using *t*-tests and Mann–Whitney test, respectively, to compare the data of two groups. Categorical variables were recorded as ratios or percentages using χ^2^ to test the significance between groups. *P*-value < 0.05 was defined as statistically significant.

The significance of alpha-diversity and beta-diversity (between sample community dissimilarity) was calculated using Kruskal–Wallis test and permutational multivariate ANOVA (PERMANOVA). Linear discriminant analysis (LDA) effect size (LEfSe) was applied to discern significant differences in the relative abundance of microbial taxa among all the groups, which included the non-parametric Kruskal–Wallis test and LDA^2^ (Segata et al., 2011). Taxonomical features with *P*-value < 0.05 and LDA effect size > 2 were regarded as significant microbial signatures. The differences in metabolic pathways were computed using the STAMP program, which could compare pairs of samples or samples organized into two or more treatment groups. The data were shown by an extended error bar plot with a corrected *P*-value < 0.05 (Parks et al., 2014). Networks analysis of microbial community and phenotype were calculated and visualized by the “Gephi” interactive platform (Ver 0.9.2) (Bastian et al., 2009; Grandjean, 2015). The networks were constructed on the correlations with absolute values of Spearman’s coefficient > 0.6 and *P* < 0.05.

## Results

### Demographic and Clinical Characteristics

A total of 177 newborns were recruited for our study, including 115 natural birth newborns and 62 cesarean section newborns. Maternal and neonatal information and medical history were obtained from medical records, namely, immune disease, PROM, and GDM. In addition, 113 pregnant women of all the participants provided abortion history and B-ultrasound reports at the same time. We defined newborns whose mothers have the immune disease, PROM, GDM, or abortion history as affected groups, and the other newborns were control groups. The immune disease included chronic urticaria and rheumatic immune disease. There were no significant differences in any of the B-ultrasound and phenotype parameters between the affected and control groups (*P* > 0.05). Demographic and clinical characteristics of newborns and mothers were summarized in Table 1.

**TABLE 1:** Demographic and clinical characteristics.

Characteristic	
**Neonatal variables**	
**Sex**	
Male	86/177 (48.6%)
Female	91/177 (51.4%)
**Phenotype**	
Length (cm)	50 (49,51)
Weight (g)	3363.9 ± 356.8
Pregnancy time (week)	39.31 ± 1.16
**B-ultrasound information**	
Time (week)	37 (37,38)
Biparietal diameter (cm)	9.21 ± 0.36
Head circumference (cm)	32.81 ± 1.06
Abdominal circumference (cm)	33.21 ± 1.50
Femur length (cm)	7.10 (6.90,7.20)
**Maternal variables**	
**Pregnancy history**	
**Mode of delivery**	
Cesarean section	62/177 (35.0%)
Natural delivery	115/177 (65.0%)
**Times of pregnancy (times)**	
1	61/113 (54.0%)
2	31/113 (27.4%)
3	14/113 (12.4%)
4	6/113 (5.3%)
5	1/113 (0.9%)
**Times of delivery (times)**	
1	84/113 (74.3%)
2	29/113 (25.7%)
**Abortion history**	
Yes	38/113 (33.6%)
No	75/113 (66.4%)
**Times of abortion (times)**	
0	75/113 (66.4%)
1	27/113 (23.9%)
2	9/113 (8.0%)
3	1/113 (0.9%)
4	1/113 (0.9%)
**Medical history of pregnant women**	
GDM	69/177 (39.0%)
PROM	38/177 (21.5%)
Immune disease	4/177 (2.3%)

*GDM, Gestational diabetes mellitus; PROM, premature rupture of membranes.*

### Difference of Oral Microbial Diversity

**FIGURE 1:**
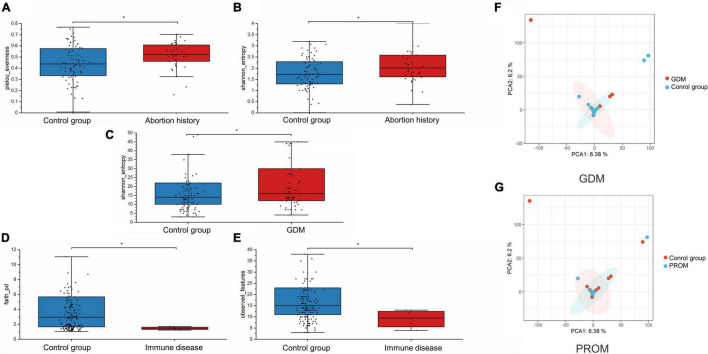
Alpha-diversity was displayed in abortion history [pielou_evenness (**A**, *P* = 0.027) and Shannon_entropy (**B**, *P* = 0.014)], GDM [Shannon_entropy (**C**, *P* = 0.035)], and immune disease [Faith_PD (**D**, *P* = 0.024] and Observed_features (**E**, *P* = 0.044) groups. PCA analysis of neonatal microbiome in GDM (**F**, *P* = 0.709) and PROM (**G**, *P* = 0.865) history groups. *P < 0.05.

We assessed alpha-diversity quantitatively by Observed_features, Shannon, Faith_PD, and pielou_evenness indices in this study. The differences between the affected and control groups were exhibited in Figures 1A–E. Among them, the alpha-diversity of pregnant women with GDM, abortion history, and without immune disease groups was higher than in control groups, indicating that a more diverse oral microbiome community structure existed in these groups (*P* < 0.05). The other alpha-diversity was not significant in our analysis (*P* > 0.05). Meanwhile, there was not a significant dissimilarity in beta-diversity between different groups (Table 2).

**TABLE 2:** Beta-diversity between different groups based on pairwise permanova results of Bray–Curtis dissimilarity.

Group	Sample Size	Pseudo-F	*P*-value
Mode of delivery	177	0.773741	0.648
Abortion history	113	1.301929	0.155
GDM	177	0.781726	0.662
PROM	177	0.612938	0.879
Immune disease	177	0.674609	0.874

We applied PCA to further visualize and discover the difference in the neonatal microbiome. A certain extent of variation was spotted between GDM, PROM, and control groups (*P* > 0.05), shown in Figures 1F,G. The samples of other groups overlapped greatly, suggesting that the two groups had similar oral microbiota structures.

**FIGURE 2:**
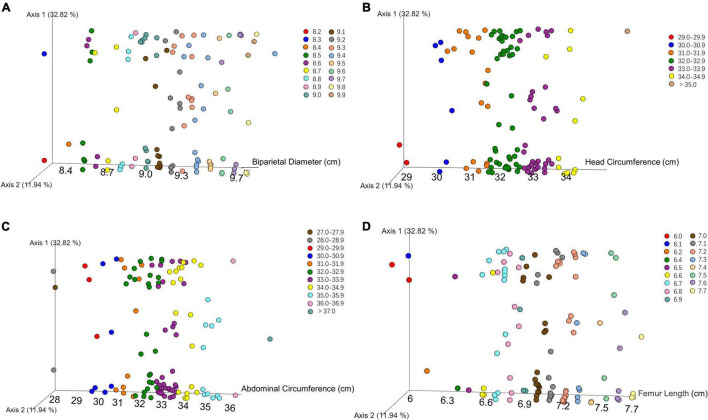
PCoA plot showed that there was no obvious separation or trend in oral microbial community among newborns with different biparietal diameters **(A)**, head circumferences **(B)**, abdominal circumferences **(C)**, and femoral lengths **(D)** using unweighted UniFrac distances.

We performed principal coordinates analysis (PCoA) on unweighted UniFrac-distance according to the B-ultrasound record. There was no obvious separation or trend with different biparietal diameters, head circumferences, abdominal circumferences, and femoral lengths in Figure 2.

### Microbial Structure of Each Group

**FIGURE 3:**
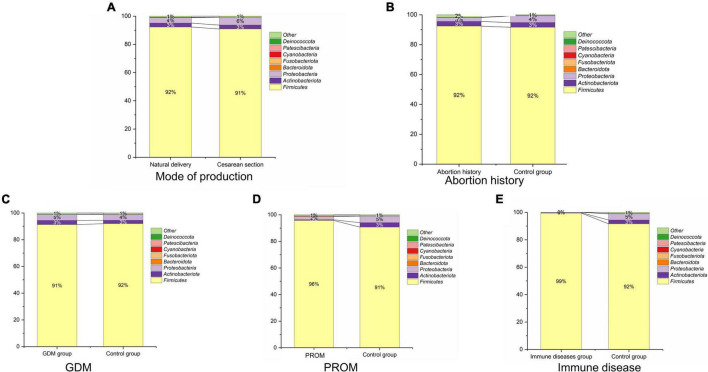
Microbial structure in mode of delivery **(A)**, abortion history **(B)**, GDM **(C)**, PROM **(D)**, and immune disease **(E)** group.

We displayed the relative abundances of the main phyla in each sample in Figure 3, which included *Firmicutes*, *Actinobacteriota*, *Proteobacteria*, *Bacteroidota*, *Fusobacteriota*, *Cyanobacteria*, *Patescibacteria* (CPR), and *Deinococcota*. Except for the abortion history group, *Firmicutes* (91.8 ± 18.7%), *Proteobacteria* (4.4 ± 13.2%), and *Actinobacteriota* (2.7 ± 10.4%) were the most predominant bacterium of neonatal oral samples among these phyla. In the abortion group, *Firmicutes* (92.4 ± 16.6%), *Actinobacteriota* (3.1 ± 10.0%), and *Proteobacteria* (2.7 ± 7.2%) were the top three phyla in bacterial abundance in Figure 3E.

### Different Genera Between Different Groups

**FIGURE 4:**
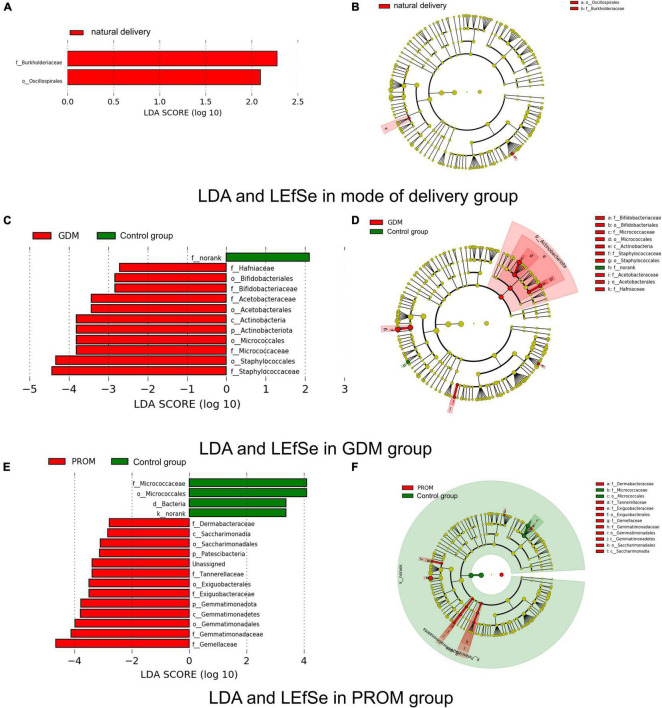
Different genera measured by LDA and LEfSe in mode of delivery **(A,B)**, PROM **(C,D)**, and GDM **(E,F)** group.

We used LEfSe and LDA to analyze and detect different genera between different groups. *Burkholderiaceae* and *Oscillospirales* were significantly more abundant in the natural delivery group than in the cesarean section group (Figures 4A,B). Significant variations were observed in *Micrococcaceae*, *Hafniaceae*, *Staphylococcaceae, Bifidobacteriaceae*, *Acetobacteraceae* in the GDM group (Figures 4C,D). *Gemellaceae*, *Saccharimonadales*, *Exiguobacteraceae*, *Gemmatimonadaceae*, *Dermabacteraceae*, and *Tannerellaceae* were enriched in the PROM group, while *Micrococcaceae* were relatively plentiful in the control group (Figures 4E,F). There were no different genera in abortion history and immune disease group based on our threshold value set.

### Different Metabolic Function Enrichment Analysis

**FIGURE 5:**
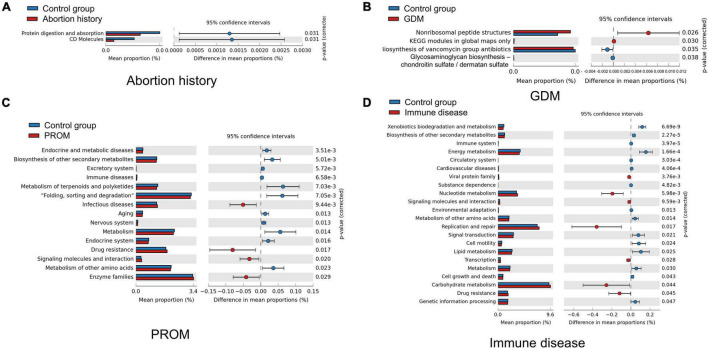
Different metabolic function enrichment analysis in abortion history **(A)**, GDM **(B)**, PROM **(C)**, and immune disease **(D)**.

We used Tax4Fun to predict the functional profiles of oral microflora, and STAMP to display differences. CD molecules, protein digestion, and absorption were significantly more abundant in the control group than in the abortion history group (Figure 5A). Non-ribosomal peptide structures, Kyoto Encyclopedia of Genes and Genomes (KEGG) modules in global maps only were enriched in the GDM group, and biosynthesis of vancomycin group antibiotics, glycosaminoglycan biosynthesis—chondroitin sulfate/dermatan sulfate was less active (Figure 5B). In PROM groups, there were significant differences in 131 KEGG Orthology (KO) pathways. For example, pathways associated with drug resistance, infectious diseases, signaling molecules and interaction, and enzyme families were more active (Figure 5C). In immune disease groups, 234 KO pathways showed a significant difference at *P* < 0.05, in which some pathways related to nucleotide metabolism, replication and repair, drug resistance, and carbohydrate metabolism were more active (Figure 5D). There was no significant difference in KO pathways in the mode of delivery group.

### Networks Analysis of Microbial Community and Phenotype

**FIGURE 6:**
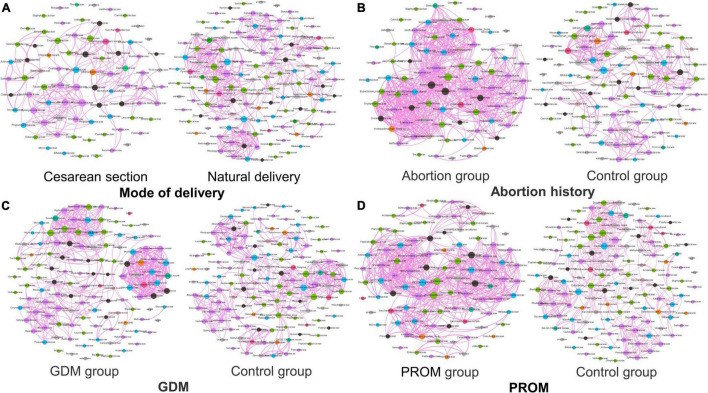
Network analysis in mode of delivery **(A)**, abortion history **(B)**, GDM **(C)**, and PROM **(D)**.

The network analysis model was used to analyze the relationship between neonatal oral microbial community and phenotype (height and weight). Networks of the mode of the delivery group (Figure 6A) were distributed evenly, while the obvious collective pattern presented in abortion history (Figure 6B), GDM (Figure 6C), and PROM (Figure 6D) groups. Also, we measured the network with various parameters for quantitative analysis, average degree, average weighted degree, diameter, average path length, and graph density (Table 3). Lower average path length and higher graph density were calculated in abortion history, GDM, and PROM groups. Due to too few samples to form the obvious aggregation, we did not perform correlation analysis in the immune disease group.

**TABLE 3:** Networks analysis of microbial community and phenotype.

Group	Average degree	Average weighted degree	Diameter	Average path length	Graph density
**Mode of delivery**					
Cesarean section	6.575	8.816	8	3.621	0.083
Natural delivery	8.064	11.796	9	3.795	0.065
**Abortion history**					
Abortion group	17.506	26.596	6	2.109	0.219
Control group	6.784	9.831	10	4.136	0.071
**GDM**					
GDM group	9.317	14.204	10	3.350	0.078
Control group	8.064	11.796	9	3.795	0.065
**PROM**					
PROM group	13.853	20.11	7	2.653	0.147
Control group	8.064	11.796	9	3.795	0.065

## Discussion

We explored and analyzed neonatal oral microbiota by 16S rRNA gene sequencing, suggesting that mode of delivery and maternal factors might have a crucial role in oral microflora. Our results encouraged positively module maternal health status to reduce the risk of neonate diseases by initial oral microbial habitat regulation.

*Firmicutes*, *Actinobacteriota*, *Proteobacteria*, and *Bacteroidota* were the most predominant phyla in our study, which was similar to the previous study (Bäckhed et al., 2015; Al-Shehri et al., 2016; Shi et al., 2018). And significantly higher abundance of *Burkholderiaceae* and *Oscillospirales* were observed in natural delivery compared with cesarean section neonates. *Oscillospira*, one genus of *Oscillospirales*, frequently detected in the human gut microbiota, was demonstrated to be intimately associated with human health, whose abundance was positively correlated with microbial diversity, high-density lipoprotein, and sleep time. *Oscillospira* could be a predictor of low BMI and constipation (Chen et al., 2020). The low abundance of *Oscillospira* might be associated with many diseases, such as type 2 diabetes, ulcerative colitis, and inflammatory bowel disease (Zhu et al., 2020; Yang et al., 2021). The cesarean section would cause physical injury and inflammatory reaction, leading to the decrease of *Oscillospira*. However, Matsuyama suggested that higher *Oscillospira* was associated with oral antibiotic exposure, such as oral antibiotics, and was born by the cesarean delivery. The reason why not find the difference in the gut microbial community from antibiotic exposure among children breastfed might be that breastfeeding might alleviate the influence of antibiotic exposure (Matsuyama et al., 2019). The mechanism of *Oscillospirales* in the mode of delivery still required to be identified.

The differences in alpha-diversity were exhibited in GDM, abortion history, and immune disease history groups. Meanwhile, the obvious collective pattern presented in networks of abortion history, GDM, and PROM groups. Our results suggested that maternal factors might be relevant to the colonization of neonatal oral microbiota. Besides, maternal infection and medical history might cause adverse pregnancy outcomes and affect the microbial community of newborns (Ishimwe, 2021). During pregnancy, maternal, and uteroplacental environments, the microbiome had a significant role in the health outcomes of the posterity through developmental programming and immune mechanisms. Also, Bearfield et al. demonstrated that there was a significant correlation between microbial DNA in amniotic fluid and complications in previous pregnancies, namely, abortion, intrauterine death, neonatal death, preterm delivery, and PROM (Bearfield et al., 2002). In addition, the current analysis of networks and PCoA did not exhibit the relationship between neonatal oral microbiota and growth phenotypes, which remained to develop.

Moreover, our study first revealed the function of abortion history on neonatal oral microbiota. Protein digestion and absorption and CD molecules were relatively inactive in the oral microbiota of newborns delivered by women with abortion history, meaning the decline in protein metabolism and immune ability compared with groups without abortion history. Abortion might be associated with inflammation in the uterus or harmful microorganisms in the cervix, such as *L. amnionii*, *A. vaginae*, and *S. sanguinegens*, disturbing the microbial structure in the maternal uterus (Seo et al., 2017), to change the microbial habitat of newborns.

We observed that chondroitin sulfate (CS) biosynthesis capabilities of neonatal oral microbe fell in the GDM group. CS, as a sulfated glycosaminoglycan (GAG), was a kind of important biological macromolecule and largely presented in the extracellular matrix, one of which was dermatan sulfate (DS). CS exhibited various biological activities, such as anti-inflammation, antioxidation, neuroprotection, and antineuroinflammation (Holgerson et al., 2013; Gao et al., 2020). The different contents and compositions of GAGs have been found in many diseases (Morla, 2019; Kwok et al., 2021; Meng et al., 2021). The total amount of GAGs and DS increased significantly in diabetes rats (Wang, 2009). The microbiota of GDM pregnant women could vertically transmit to the offspring (Wang et al., 2018). In our research, the falling capabilities of CS biosynthesis of microbe might compensate for the increased amount of CS in the organism to regulate human health.

The first limitation of our study was the lack of a longitudinal follow-up study of infant oral samples. Secondly, our study lacked the collection of the maternal vagina, amniotic fluid, and skin samples, which had a considerable impact on the infant microbial community. In addition, the number of pregnant women with specific medical history was few, causing a deviation in experimental results. Only four pregnant women with immune diseases were enrolled in our research. Moreover, we recorded other medical history and analyzed microbial diversity, including scar uterus, liver disease, and hypertension. No significant difference in those groups was measured. However, we did not list the part of the results in our article. In the future, we would increase the enrollment and explore whether these systemic diseases affect the oral microbial composition of newborns. Finally, many factors such as maternal diet, prenatal weight, and antibiotics exposure might affect the colonization of neonatal microbe, not allowing us to correct for common confounders.

## Conclusion

In summary, our study supplied further evidence that the influence of maternal factors and mode of delivery on neonatal oral microbiota, namely, abortion history, GDM, PROM, and immune disease, whose diversity, metabolic function, and community structure might change. Furthermore, a higher abundance of *Oscillospirales* was observed in neonates of vaginal birth. The current analysis did not show the correlation between neonatal oral microbiota and development phenotypes. Our findings could enhance our awareness of modulations of oral microbial composition to provide a lush landscape for the prevention of neonatal diseases with potentially novel mechanisms of action.

## Data Availability Statement

The data presented in this study are deposited in online repository. The names of the repository/repositories and accession number(s) can be found below: https://ngdc.cncb.ac.cn/gsa/, CRA006476.

## Ethics Statement

The studies involving human participants were reviewed and approved by the Conjoint Health Research Ethics Board of Peking University People’s Hospital. Written informed consent to participate in this study was provided by the participants’ legal guardian/next of kin.

## Author Contributions

LY, BS, QX, and JZ: sample collection and DNA extraction. BS and QX: clinical sample collection. TX, WZ, and QZ: statistical and sequencing analyses and management. TX, LY, FC, and GL: manuscript writing. NC, FC, and GL: project supervision and design. All authors contributed to the article and approved the submitted version.

## Conflict of Interest

The authors declare that the research was conducted in the absence of any commercial or financial relationships that could be construed as a potential conflict of interest.

## Publisher’s Note

All claims expressed in this article are solely those of the authors and do not necessarily represent those of their affiliated organizations, or those of the publisher, the editors and the reviewers. Any product that may be evaluated in this article, or claim that may be made by its manufacturer, is not guaranteed or endorsed by the publisher.

## Footnotes

1
http://qiime.org/

2
http://huttenhower.sph.harvard.edu/lefse/
